# Integrating Top-down and Bottom-up Requirements in eHealth Development: The Case of a Mobile Self-compassion Intervention for People With Newly Diagnosed Cancer

**DOI:** 10.2196/37502

**Published:** 2022-08-01

**Authors:** Judith Austin, Constance H C Drossaert, Jelle van Dijk, Robbert Sanderman, Elin Børøsund, Jelena Mirkovic, Marijke Schotanus-Dijkstra, Nienke J Peeters, Jan-Willem J R Van 't Klooster, Maya J Schroevers, Ernst T Bohlmeijer

**Affiliations:** 1 Section of Psychology, Health & Technology Faculty of Behavioural, Management and Social Sciences University of Twente Enschede Netherlands; 2 Faculty of Engineering Technology University of Twente Enschede Netherlands; 3 Department of Health Sciences University of Groningen University Medical Center Groningen Groningen Netherlands; 4 Department of Digital Health Research Division of Medicine Oslo University Hospital Oslo Norway; 5 Behavioural Management and Social Sciences Lab Faculty of Behavioral, Management and Social Sciences University of Twente Enschede Netherlands

**Keywords:** eHealth, cancer, self-compassion, co-design, requirements, evidence-based, mobile phone

## Abstract

**Background:**

Psychosocial eHealth interventions for people with cancer are promising in reducing distress; however, their results in terms of effects and adherence rates are quite mixed. Developing interventions with a solid evidence base while still ensuring adaptation to user wishes and needs is recommended to overcome this. As most models of eHealth development are based primarily on examining user experiences (so-called *bottom-up* requirements), it is not clear how theory and evidence (so-called *top-down* requirements) may best be integrated into the development process.

**Objective:**

This study aims to investigate the integration of top-down and bottom-up requirements in the co-design of eHealth applications by building on the development of a mobile self-compassion intervention for people with newly diagnosed cancer.

**Methods:**

Four co-design tasks were formulated at the start of the project and adjusted and evaluated throughout: *explore* bottom-up experiences, *reassess* top-down content, *incorporate* bottom-up and top-down input into concrete features and design, and *synergize* bottom-up and top-down input into the intervention context. These tasks were executed iteratively during a series of co-design sessions over the course of 2 years, in which 15 people with cancer and 7 nurses (recruited from 2 hospitals) participated. On the basis of the sessions, a list of requirements, a final intervention design, and an evaluation of the co-design process and tasks were yielded.

**Results:**

The final list of requirements included intervention content (eg, major topics of *compassionate mind training* such as psychoeducation about 3 emotion systems and main issues that people with cancer encounter after diagnosis such as regulating information consumption), navigation, visual design, implementation strategies, and persuasive elements. The final intervention, *Compas-Y*, is a mobile self-compassion training comprising 6 training modules and several supportive functionalities such as a mood tracker and persuasive elements such as push notifications. The 4 co-design tasks helped overcome challenges in the development process such as dealing with conflicting top-down and bottom-up requirements and enabled the integration of all main requirements into the design.

**Conclusions:**

This study addressed the necessary integration of top-down and bottom-up requirements into eHealth development by examining a preliminary model of 4 co-design tasks. Broader considerations regarding the design of a mobile intervention based on traditional intervention formats and merging the scientific disciplines of psychology and design research are discussed.

## Introduction

Receiving a cancer diagnosis and undergoing treatments can disrupt many aspects of a person’s life, often affecting not only one’s physical but also one’s mental and social well-being [1-6]. Psychological interventions for people with cancer are effective in reducing symptoms of distress and improving well-being and are mostly delivered face to face in an individual or group format [7-10].

Although face-to-face interventions may offer important benefits such as live social support, they are often not adopted by people with cancer. People with cancer already face many demands, including medical appointments. Reasons for not participating in available interventions include the burden of travel, too many competing demands, and not feeling well enough to join sessions [11]. Interventions delivered through technologies such as eHealth may offer unique benefits such as increased accessibility and scalability [12], thereby reaching people who may not have otherwise participated. In addition, offering interventions in a mobile format may help with the integration of newly learned skills into daily life, as most people currently carry their mobile devices with them during daily activities [13].

Although eHealth interventions appear to be similarly effective in reducing mental distress compared with traditional intervention formats [14,15], results regarding the effects of psychological eHealth interventions in the context of cancer are still mixed [16,17], with varying rates of adherence [18]. Particularly when it comes to mobile interventions, many lack a solid foundation of theory and evidence [19]. More theory- and evidence-driven interventions are recommended for improving effectiveness and adherence [17,19-22]. Simultaneously, it is important to take into account the wishes, needs, and daily life of people with cancer to increase the chance that the intervention is successfully adopted by the target group [23,24]. Thus, what is needed to facilitate intervention success is an integration of both theory and evidence-based requirements (which we will call *top-down*; ie, from the *abstract* sphere of theory and evidence down to *concrete* experiences of daily life) and the experience-based requirements of people with cancer (which we will call *bottom-up*; ie, going from *concrete* experiences of daily life up to *abstract* theory and evidence).

This integration of top-down and bottom-up requirements may be facilitated by co-design. Co-design is a collaborative creative process through which members of the target group and stakeholders become active participants in intervention design rather than mere reactive subjects of user-centered design. In co-design, the user is not a passive object of study through only observations or interviews but an expert in their experience, with the researcher as a facilitator [25,26]. Top-down requirements could be introduced into the co-design process by researchers or other experts. Although in medical and behavioral research, top-down requirements for interventions are common [27,28], existing frameworks of eHealth development are predominantly based on bottom-up requirements and user-centered design (see the review by van Gemert-Pijnen et al [29] for an overview). Thus, it is unclear how top-down requirements can be optimally integrated into the co-design process. Without proper integration, a problematic outcome could be that an intervention has content and design that people like to use but no ground in scientific evidence. Another problematic outcome could be an application in which scientific evidence dominates the final solution, whereas experience-based requirements (gathered early on in a project) are neglected or overruled by the project team. Therefore, our overall objective is to use the co-design process to have top-down and bottom-up requirements and stakeholders explicitly meet and engage in a design conversation, leading to a coherent, integrated intervention that acknowledges the value of both types of requirements.

To meet this objective, we built on the case of the co-design of a mobile self-compassion intervention for people with newly diagnosed cancer. Although most of the discussed psychological interventions for people with cancer are based on cognitive behavioral techniques or mindfulness, compassion-based interventions for people with cancer are rapidly emerging [30]. These interventions focus on developing a compassionate acceptance of one’s distress and the motivation to alleviate the distress. Participants of various compassion-based interventions have reported increased acceptance of their illnesses and limitations, improved emotion regulation skills, and reduced feelings of isolation [30], making this type of intervention particularly relevant in the context of cancer. Indeed, our initial pilot study showed that people with cancer evaluated self-compassion as important and preferred to receive an intervention shortly after diagnosis in the form of a smartphone app [31].

Thus, the aim of our co-design study was to create an eHealth intervention that is grounded in both (1) theory and evidence-based requirements (eg, founded by established compassion-based interventions such as *compassionate mind training* [32]; ie, top-down requirements) and (2) experience-based needs, wishes, and requirements of people with cancer and oncology nurses (ie, bottom-up requirements). To achieve this integration, a set of co-design tasks were devised and evaluated throughout the development process. The co-design study yielded (1) a list of integrated top-down and bottom-up requirements, (2) a final design of a mobile self-compassion intervention for people with newly diagnosed cancer, and (3) an evaluation of the co-design process and tasks. On the basis of these outcomes, we will discuss the potential relevance of our co-design approach as a preliminary model for integrating top-down and bottom-up requirements in eHealth development.

## Methods

### Study Design

As recommended by the Centre for eHealth Research Roadmap approach to eHealth development, the design and development process constituted a participatory approach using continuous cycles of evaluation [29]. Throughout this process, co-design methods were used in which people with cancer and oncology nurses served as the experts in their experiences [25]. The study was led by a project team comprising researchers with a background in either psychology (including health psychology and compassion science) or design, as well as patient advisers, oncologists, clinical psychologists, and software developers.

### A Priori Outline of Co-design Tasks and Top-down Requirements

Informed by existing eHealth development frameworks (see the review by van Gemert-Pijnen et al [29]), we adapted our approach to explicitly focus on the integration of top-down and bottom-up requirements. Accordingly, an outline of co-design tasks was formulated by the project team at the start of the project and adapted throughout the development process, resulting in the following four iterative co-design tasks: (1) *explore* bottom-up experiences, (2) *reassess* top-down content, (3) *incorporate* bottom-up and top-down input into concrete features and design, and (4) *synergize* bottom-up and top-down input into the intervention context (Textbox 1 provides an overview). These tasks were executed during a series of co-design sessions, as described in the following sections. In addition, to explore in-depth personal accounts of experiences with self-compassion after diagnosis, the development of intervention content was conjointly informed by semistructured individual interviews with people with cancer [33].

**The 4 iterative co-design tasks to enable the integration of top-down and bottom-up requirements.**

**Co-design task and description**

1. *Explore* bottom-up experiences
  - Acquire input on experienced challenges and facilitators (in general and in relation to top-down scope) and the most important targets and topics for the intervention according to participants.
2. *Reassess* top-down content
  - Assess top-down content in the context of user recognition, appreciation, and suggestions for alterations.
  - Make adaptations to top-down content according to the needs and vocabulary of users and reframe user wishes based on top-down content.
3. *Incorporate* bottom-up and top-down input into concrete features and design
  - Specify and integrate bottom-up and top-down requirements by translating them into concrete features and design and then tangibly explore similarities and differences.
  - Assess which bottom-up features are put forward by participants and how participants experience features derived from top-down requirements.
  - Discuss and prioritize requirements (using co-design exercises and trade-off decision-making strategies).
4. *Synergize* bottom-up and top-down input into the intervention context
  - Focus on synergizing requirements into all levels of the intervention context.
  - Match the overall structure of the intervention (eg, ordering, logic, and main interface), communication channels (eg, level of external support), and interaction flow to both the top-down requirements (eg, regarding intervention rationale and implementation factors) and bottom-up requirements (eg, regarding routines and life patterns of the user and stakeholders).

Before the start of the co-design sessions, top-down requirements were formulated for the self-compassion intervention based on existing compassion theory and evidence on compassion-based interventions (for an overview of intervention elements and evidence of effectiveness, see the review by Austin et al [30]), as well as on the characteristics of effective eHealth interventions. Compassionate mind training served as the main framework for the intervention, which uses an evolutionary-based model of 3 emotion systems, and focuses on understanding our minds and emotions, developing feelings of compassion (including for experienced self-criticism) and compassionate acceptance, and developing skills such as mindful awareness and compassionate imagery [32,34,35]. There is increasing evidence that compassionate mind training, offered as part of compassion-focused therapy or in a nonclinical form, is effective in improving well-being and reducing distress in general populations and populations with chronic illness [30,36-38]. In addition, exercises from positive psychology and *Mindful Self-Compassion Training* [39] were included in the development process. Furthermore, characteristics that are known to promote the effectiveness of and adherence to eHealth interventions were considered top-down requirements, particularly persuasive design principles such as self-monitoring, receiving rewards, and social support [40]. In addition to compassion-based intervention content, compassion as a design value was considered a top-down design requirement. In face-to-face compassion training or therapy, the trainer models compassion throughout the training process (ie, with compassionate responses to difficulties and deshaming of experiences) [41]. An aim of the development process was to model compassion throughout different features and contents of the intervention (eg, providing compassionate feedback when a user indicates high levels of distress). Taken together, this input formed the theoretical starting point for the co-design sessions (ie, it provided the general frame and scope of the challenge to be explored with participants) and was introduced during various co-design exercises, particularly in the task *reassess top-down content* (Table 1 provides an overview of these co-design exercises).

**Table 1:** Overview of sessions, co-design exercises, and co-design tasks.

Sessions and co-design exercises		Co-design task
**Session 1**		
	Mapping of individual obstacles and facilitators in dealing with the cancer diagnosis, visualized as rocks and ladders	Explore
	Mapping of support that was or was not present from oneself, own network, or professionals after the diagnosis using a card sorting method	Explore
	Identifying individual moments of self-compassion and self-criticism on sticky notes in relation to the diagnosis and then categorizing them together	Explore
**Session 2**		
	Trying out self-compassion exercises in the 2 weeks before the session; building a desired app and an undesired app represented on paper smartphone models by categorizing and altering the self-compassion exercises	Reassess
	Identifying additional topics and exercises to be addressed in the app by adding to and altering topics identified in the first session	Explore
**Session 3**		
	Trying out other psychosocial apps in the week before the session; presenting the apps in small groups, highlighting positive and negative user experiences; creating a map of the similarities and differences in the experiences of functionalities in these apps, focused on filling out and sharing information, motivational elements, feedback, personalization, and mode of information	Integrate
	Exploring language use in the app by playing a card game in which the story of the app was presented in 5 different ways (based on metaphors) on 5 cards, where participants “played out” their preferences	Integrate
	Creating a diagram of the way the app could be offered and supported by nurses (when, to whom, how, and how often)	Synergize
**Session 4**		
	Shaping the flow of and processes within the app using cardboard boxes representing different app modules to write on and move around	Synergize
	Creating paper prototypes of parts of the app using both defined (eg, printed buttons) and undefined (eg, random or blank stickers) materials	Integrate
**Session 5**		
	Interacting with a low-fidelity prototype of a home page and engaging with different home page designs represented on posters	Integrate
	Role-plays around app implementation and app recommendation by nurses and people with cancer	Synergize
	Interacting with a low-fidelity prototype of the content of an app module in the form of a smartphone app, as well as on paper	Reassess
**Session 6**		
	Refining wireframes and high-fidelity prototypes provided by the app developer (also in participants’ home settings)	Integrate
	Mapping implementation processes and challenges based on diagrams from session 3 (nurses only)	Synergize
	Generating ideas for peer tips and experiences to be included in the app in a card-based group game	Explore
**Session 7**		
	Evaluating the “final” version of the intervention in terms of bottom-up requirements (with minor changes still implemented) using whiteboards	(Evaluate)
	Evaluating the co-design process using interview methods among participants	(Evaluate)

### Participants and Procedures

Participants for the co-design sessions were recruited from 2 participating hospitals (1 community and 1 university hospital). Eligible participants were adults with any form of cancer diagnosed 6 to 24 months ago who were willing to participate in ≥1 session and had sufficient command of the Dutch language. In addition, oncology nurses who work with people with cancer were selectively recruited by the project’s consulting oncologists. People with cancer received a study information leaflet from oncology nurses during regular consultations, which contained an overview of the study procedures and referred them to the study website for information about the study, privacy regulations, contact opportunities, and sign-up. A total of 15 people with cancer (n=8, 53% female, and n=7, 47% male; aged 29-64 years), who were diagnosed 6 to 24 months ago with a form of cancer (breast cancer n=6, 40%; all other forms n=1, 7% each), and 7 oncology nurses (n=4, 57% female, and n=3, 43% male; aged 31-54 years) with 9 to 28 years of experience working with people with cancer were included.

### Ethics Approval

Written informed consent was obtained from all participants at their start of the first co-design session. Consent for visual recording (photo or video) was reconfirmed on each occasion (verbal or written). This study was approved by the Ethical Committee BMS of the University of Twente (approval number BCE18853).

### Co-design Sessions

A total of 7 rounds of co-design sessions were conducted over a period of 2 years (November 2018 to November 2020). In each session, 2 to 3 oncology nurses, 3 to 6 people with cancer, and 2 to 3 facilitating researchers were present. The 7 sessions were conducted twice, with participants from 2 distinct geographical areas (ie, the 2 hospitals), once at a university medical center and once at a university. Each session lasted between 2 and 3.5 hours (session duration was adapted based on the energy levels of participants). The last round of sessions was delayed and partially conducted on the web because of the COVID-19 pandemic. Sessions generally comprised an introduction, with a recap of the previous session, followed by an icebreaker creative exercise, 2 to 3 co-design exercises, a general discussion, and a concluding evaluation questionnaire. A mix of group and individual co-design exercises was used to foster creative idea generation [42,43]. A variety of co-design exercises was used, which could be categorized as *making tangible things* (eg, creating 2D maps and prototyping); *talking, telling, and explaining* (eg, card sorting and group discussions); and *acting, enacting, and playing* (eg, group games and role-play) [43]. In contrast to user-centered design approaches in which user input is analyzed by researchers behind the scenes, input from the exercises was discussed, prioritized, and summarized during the co-design sessions as much as possible, ensuring participants’ active role in the interpretation of the results. Small group exercises were conducted with people with cancer and nurses separately, after which the outcomes were integrated into collective discussions and exercises. This approach was chosen to benefit from multidisciplinary perspectives while also creating a safe environment to share experiences among peers. In addition, participants occasionally engaged in exercises between sessions in their home environment (eg, usability testing of high-fidelity prototypes). Data were collected using physical materials from the co-design tasks (eg, paper maps and sticky notes), as well as audio recordings, written notes, and occasional video recordings.

**Figure 1:**
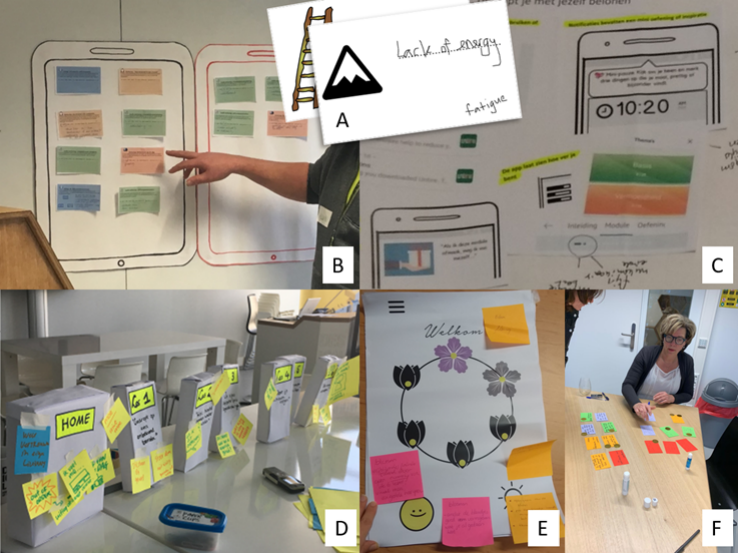
Examples of paper materials used in the co-design exercises. The co-design exercises are described in Table 1 . (A) Obstacle card (session 1, first exercise). (B) Desired and undesired apps (session 2, first exercise). (C) Map of motivational elements (session 3, first exercise). (D) Cardboard boxes representing the app modules (session 4, first exercise). (E) Poster of a home page design (session 5, first exercise). (F) Card game about the tips (session 6, third exercise).

The 4 co-design tasks were used iteratively across sessions rather than only sequentially, thus encompassing the components of different sessions. Study-specific session evaluation questionnaires addressed satisfaction, burden, inspiration, collaboration, learning new things, alignment with personal expertise, sense of involvement with the project, and sense of influence over the design on a 5-point Likert scale, with room for open-text input (eg, “To what extent do you feel involved with developing a self-compassion app for people with cancer?”; Multimedia Appendix 1). During the last session, the full co-design process and final design were evaluated with participants. Table 1 provides an overview of sessions, co-design exercises, and tasks, and Figure 1 presents visual examples of paper materials used in the co-design exercises.

### Integrating Top-down and Bottom-up Requirements

The final requirements were yielded from the 4 co-design tasks, during which initial ideas for requirements were processed and prioritized (based on the MoSCoW categorization of *must haves* and *nice to haves* [44]). Trade-off decision-making was used to balance various (conflicting) requirements. For example, a top-down requirement was to include caregiver support within the app to increase intervention effectiveness [37,45], whereas bottom-up requirements were to minimize the workload of nurses and have a private intervention experience for people with cancer (see the study by Austin et al [46] for more details and examples of the strategies we used to merge conflicting requirements). Following the completion of a provisional list of requirements (session 4), collaboration with a commercial app developer agency was initiated. Financial and technical opportunities and constraints were then taken into account in the further prioritizing and refinement of requirements. Although some of the processing of requirements was done by the project team in between sessions (eg, gaining an overview of the financial impact of different requirements), most of the prioritization was done during co-design sessions in collaboration with participants (ie, with the aforementioned co-design exercises). The final requirements included functional (ie, what the intervention should do) and nonfunctional (ie, properties of the intervention such as usability) requirements [47]. Detailed software requirements (eg, “when user clicks X, Y should appear”) were derived from the final requirements and are beyond the scope of this paper, as are specific formatting and visual design issues.

## Results

In this section, we describe the outcomes of the seven co-design sessions: (1) the final list of requirements for the intervention and how they were implemented; (2) the content and functionalities of the intervention; and (3) an evaluation of the co-design process, particularly the 4 co-design tasks. Evaluation and implementation of the intervention were not part of this study.

### Final Requirements

Table 2 summarizes the main list of requirements. These requirements included aspects such as the content of the app (eg, psychoeducation about the 3 emotion systems tailored to the context of cancer), navigation (eg, having the option to skip or save exercises), visual design (eg, minimal and soothing), implementation (eg, a stand-alone app embedded in regular care), and persuasive elements (eg, receiving push notifications). The final requirements were the direct outcomes of the co-design sessions. For example, a co-design exercise addressing obstacles and facilitators after diagnosis (session 1, first exercise; Table 1 ) yielded experienced obstacles of a lack of energy and mental clarity. Furthermore, it became clear across sessions that participants would value help in remembering to engage with the app and staying motivated, without feeling pressured. Specifically, evaluating other apps (session 3, first exercise; Table 1 ) showed that subtle motivational elements in the form of viewing progress within the app or receiving inspirational messages (eg, a progress bar that changes color and a tip of the day) would be fitting, in contrast to earning badges or points: “You already feel miserable, you shouldn’t have to earn anything. But there has to be something that pulls you to the app.” Motivational elements were then further examined by prototyping (eg, session 4, second exercise; session 6, first exercise; Table 1 ). On the basis of these outcomes of various co-design exercises, requirement 8 regarding subtle persuasive elements was formed. Although these requirements are specific to this intervention, generalizable intervention characteristics may be inferred from each. For instance, the abovementioned example illustrates that noninvasive and inspirational persuasive design elements are implemented to make the intervention engaging without being perceived as inappropriate or coercive (eg, notifications containing a quote or brief exercise rather than an explicit reminder to complete an exercise). Similarly, in balancing tunneled versus freely available content, requirement 15 illustrates that we implemented both types of content, which were then cross-referenced (eg, pointing out relevant module content in the automated feedback of the mood tracker; a freely accessible exercise *light of the day* is expanded upon in 2 modules).

Following prioritizing and trade-off decision-making, all the main requirements were met in the intervention design. However, some functionalities were implemented in a simplified form, and some requirements were only partially met. For example, we included a mix of audio, video, text, and images to convey information; however, we were unable to include audio recordings for all written text to listen to instead of reading. The participants indicated that this would substantially help with concentration difficulties; however, financial constraints prevented us from implementing this. Textbox 2 presents an overview of the ways in which the final requirements (as listed in Table 2) were implemented in the intervention.

**Table 2:** Final list of matched top-down and bottom-up requirements.

Top-down requirements	Bottom-up requirements
1. Linking existing content of compassionate mind training to bottomup challenges to create a tailored intervention	1. Topics to include in the intervention: accepting the illness and limitations, taking care of one’s body, asking for and accepting help, guarding social and physical boundaries, motivating oneself in a positive way, coping with anxiety, and regulating information consumption
2. Main focus on self-compassion training that can be applied to various practical contexts	2. Receiving ample, practical, and localized information about the treatment of and living with cancer
3. Psychoeducation about 3 emotion systems, self-compassion, and selfcriticism	3. To-the-point and practical psychoeducation tailored to the context of cancer
4. Reflective exercises about 3emotion systems, self-compassion, and self-criticism	4. Exercises that generate insight into and awareness of emotions and self-talk in the context of cancer
5. Mindfulness exercises, soothing rhythm breathing, and visualization exercises	5. Brief meditative exercises with down-to-earth, nonspiritual language that facilitate rest
6. Having compassion for one’s distress (offering compassionate feedback) and training own capacity to notice and reduce distress	6. Tips and tricks to “get rid of” distress (eg, in automated feedback)
7. Address all key elements of compassionate mind training, adapted from traditional intervention formats	7. Mix between “bite-sized” text, video, images, and audio to convey information (to help with concentration difficulties)
8. Persuasive design elements such as rewards and praise	8. Subtle motivational elements without too much gamification
9. Mood tracking to enhance awareness of emotions and facilitate compassionate responding	9. Mood tracking on multiple scales, having an overview of mood changes over time, and optional feedback
10. Use social support persuasive design elements such as social facilitation	10. Having a private app without direct peer contact while including experiences of peers
11. Pseudonymous rather than anonymous app use to collect research data (ie, creating a user account)	11. Onboarding and log-in process as simple and fast as possible while safeguarding privacy
12. Visual design that aligns with self-compassion training	12. Minimal and soothing visual design
13. Appealing to and reaching a broad range of people in a low-threshold way	13. Appealing to and reaching a broad range of people in a low-threshold way
14. Support of health professionals with(in) the app	14. Stand-alone private app for users, which does not create extra workload for nurses
15. Sequential, modular learning structure	15. Freedom to navigate to any relevant content (including skipping or saving content)

**Overview of implementation of the final list of requirements into the intervention.**

**Implementation of requirements into the intervention**

1. Each main module addresses a main element from compassionate mind training; all such elements are explained in the context of bottom-up topics; submodules address different bottom-up topics
2. The intervention has a main focus on self-compassion training adapted to the context of cancer; an information page contains selected weblinks with practical cancer-related information
3. Psychoeducation about 3 emotion systems, self-compassion, and self-criticism tailored to the context of cancer and contains practical examples
4. Reflective exercises about 3 emotion systems, self-compassion, and self-criticism tailored to the context of cancer.
5. Brief mindfulness, soothing rhythm breathing, and visualization exercises with down-to-earth, practical guidance
6. Automated feedback using compassionate language (eg, recognizing distress, acknowledging that it is part of life) that stimulates self-regulation while also offering suggestions for exercises
7. Mix between images, videos, and audio to convey psychoeducation and exercises; the use of audio is limited to meditative exercises.
8. Subtle use of rewards and praise such as receiving a visual reward upon completing a module (eg, a new part of an incomplete image appears)
9. Mood tracker on 3 scales based on the 3 emotion systems, with an option to receive automated feedback and a graph showing mood progression over time
10. A private app without direct peer contact, with quotes from peers about their experiences related to the module theme
11. Simple onboarding that requires creating an account on registration while staying logged in for subsequent sessions
12. Minimal app design using a monochromatic color scheme
13. Nurses explain the app to people with cancer in their own words, emphasizing parts of the intervention that they expect to align with their needs
14. Stand-alone private app for users without in-app communication or information sharing with nurses, integrated into regular care
15. The app contains 6 modules that can be accessed after 1 week without having completed previous content; functionalities that are freely accessible at any time from the menu bar; option to mark pages as favorite

### The Intervention: Compas-Y

**Figure 2:**
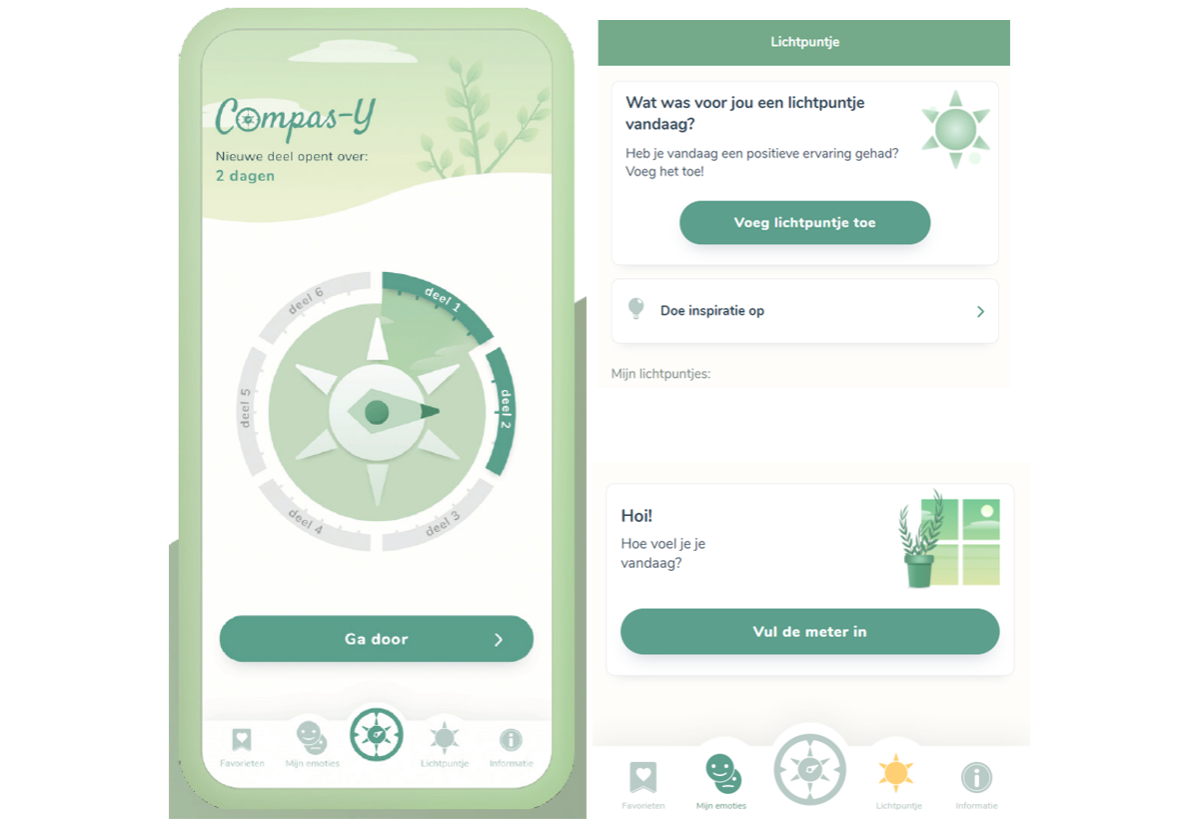
Screenshots of the Compas-Y intervention. On the left, the home page of the Compas-Y intervention containing a central compass navigation element with 6 modules and a menu bar with supportive functionalities. On the top right, the start of the exercise light of the day; on the bottom right, the start of the mood tracker.

The final mobile self-compassion intervention, *Compas-Y*, which resulted from the co-design sessions, comprises 6 sequential training modules and features that are accessible at any time from the home page. The intervention content is based on compassionate mind training, with a few additional elements of positive psychology (eg, functionality *light of the day*) and mindful self-compassion (eg, exercise “How would you treat a friend?”). Diversity, equity, and inclusion design aspects (see the study by Ramos et al [48]) were addressed to some extent, for example, by offering content that is understandable to people with various degrees of exposure to formal education, alternatives to breath-focused exercises, closed captions for videos, and diversity in visual representation. Textbox 3 provides a brief overview of the intervention content, and Multimedia Appendix 2 and Multimedia Appendix 3 [40] provide an extensive overview, including aims and user outcomes, and persuasive design elements, respectively. Each module has a theme (eg, recognizing and regulating emotions or taking care of your body) and includes psychoeducation and exercises aimed at cultivating self-compassion after a cancer diagnosis. Each module contains an optional component in which users can read the experiences (brief quotes) of peers and nurses related to the module theme. Each week, a new module becomes available regardless of the user’s progress. Features that are directly accessible from the app’s home page include a mood tracker, an exercise in which the user recalls a pleasant experience of the day (*light of the day*), a page with favorite exercises, and a practical information page. In both the module exercises and the mood tracker, automated feedback is provided based on user input (eg, “Your drive system is active. Perhaps you are feeling restless and rushed. Sometimes that is just the way it is. To not blow up this feeling, you could activate the soothing system. For example by taking a moment of rest, or by taking three deep breaths.”). Users can track their progress on the home page, where a compass symbol indicates which (components of) modules are completed, as well as which component was last opened. Push notifications are used to stimulate the integration of content into daily life. All content remains available after the intervention period of 6 weeks, and users can continue to use their favorite exercises, receive notifications, and restart the modules. Figure 2 presents screenshots of the *Compas-Y* intervention, and Multimedia Appendix 4 and Multimedia Appendix 5 present a video demonstration and additional screenshots per requirement, respectively.

**Overview of app modules and supportive functionalities with their key components.**

**Module topics and key components**

1. Introduction to the app and self-compassion
  - Psychoeducation about self-compassion
  - Exercises in mindful awareness and soothing breathing rhythm
  - Exercise in finding (brief) positive experiences throughout the day
2. Emotions in the context of cancer
  - Psychoeducation on 3 emotion systems (soothing, drive, and threat)
  - Soothing breathing rhythm exercise with imagery (soothing)
  - Compassionate information seeking; finding resources based on own needs (drive)
  - Psychoeducation about anxiety; practicing to recognize and allow anxiety (threat)
3. Self-compassion and self-criticism
  - Psychoeducation about self-compassion and self-criticism
  - Imagery exercises about compassionate self and inner critic
  - Soothing breathing rhythm exercise with a compassionate friend
  - Self-compassion; expressive writing exercise
4. Taking care of your body
  - Soothing breathing rhythm–based compassionate body scan
  - Psychoeducation and exercises about the difference between compassionate motivation and self-correction and self-critical motivation or attacking in the context of health and lifestyle behaviors
  - Psychoeducation about compassion for own needs in the context of sexuality and intimacy
5. The people around you
  - Psychoeducation about the 3 flows of compassion
  - Soothing breathing rhythm–based loving-kindness meditation
  - Setting boundaries and asking for help based on compassion for own needs
6. Continuing with resilience
  - Psychoeducation and exercises about positive psychology: gratitude, savoring, and strengths
  - Reflection on self-compassion practice and how to continue
  - Soothing breathing rhythm meditation with a focus on tone of voice and posture

**Supportive functionalities**

- Overview of modules: visual element central to the home page (compass symbol) that depicts the (availability of) 6 modules and user progress
- Mood tracker: mood tracking (1 question for each emotion system) with automated feedback based on 3 emotion systems
- Favorite exercises: marking exercises as favorite within the modules, which then appear in the user’s personal list of favorites
- Light of the day: exercise where the user inputs a (brief) positive experience of their day, supported by examples
- Practical information: list with weblinks about (living with) cancer, each with descriptions
- Push notifications: daily messages containing quotes and brief exercises, with an option to reduce the frequency or turn notifications off

### Evaluation of the 4 Co-design Tasks and Co-design Process

#### Evaluation of the 4 Co-design Tasks

Given that we were able to meet most requirements, our co-design experiences indicated that the 4 co-design tasks were successful in enabling the integration of top-down and bottom-up requirements. Throughout the development process, the co-design tasks helped address 2 main challenges. The first was the need to continuously balance integrating top-down content into the co-design exercises without being too leading. The *explore* bottom-up experiences task was helpful in examining any challenge or beneficial experience after diagnosis, as experienced by people with cancer and not only in the context of self-compassion. In contrast, the co-design exercises of the *reassess* top-down content task had the explicit goal of introducing top-down content. Thus, having 4 tasks each with its own function enabled us to give space to both types of requirements without overly prioritizing one or the other.

The second challenge that the co-design tasks helped address was prioritizing and dealing with conflicting top-down and bottom-up requirements (see the study by Austin et al [46] for our decision-making strategies). The tasks to *incorporate* bottom-up and top-down input into concrete features and design and *synergize* bottom-up and top-down input into the intervention context enabled the prioritization of requirements together with participants. This was done with explicit discussions but also by materializing the various requirements (eg, with paper prototypes), which allowed us to make conflicts and priorities tangible. These co-design exercises often quickly clarified which requirements were nonoptional (eg, bottom-up: not adding to the workload of nurses by offering a guided app; top-down: having some extent of a sequential learning structure). Thus, the 4 co-design tasks served as a guiding framework while investigating and merging different top-down and bottom-up requirements.

#### Evaluation of the Co-design Process

The 7 sessions were consistently positively received by participants, with a median score of 4 (scale of 1-5) for all workshops and evaluation questions. Thus, the sessions were well aligned with the energy levels and personal expertise of the participants and offered them inspiration and learning. Many participants particularly appreciated sharing experiences with each other and collaborating with both nurses and people with cancer to learn from and incorporate different perspectives. One of the participants described the following:

[The sessions] showed me how important such an app is, since so many people experience the same things. Yes each in their own way, but in the end quite similar.

Some participants particularly valued working together in a guided creative process to help future people with cancer, whereas others emphasized personal benefits such as increased acceptance of their illness. One of the participants described the following:

Every session was surprising [...] in the beginning I always thought, I have no idea what to put on paper, but at the end of the day we looked back and it was special to see what we came up with.

In retrospect, participants reported a sense of pride in the final design, and in it, they recognized the implementation of most of their expressed wishes and needs.

## Discussion

### Outline

This study aimed to explore ways in which top-down and bottom-up requirements can best be integrated into eHealth development by building on the case of the development of a mobile self-compassion intervention for people with newly diagnosed cancer. We discuss (1) the final requirements and the design outcome—*Compas-Y*—both as a testament to the apparent successful integration of bottom-up and top-down requirements and as an example of adapting a traditional intervention to the context of mobile technology and (2) the process of integrating top-down and bottom-up requirements using our 4 co-design tasks. Furthermore, the interdisciplinary aspects of this study, its strengths, and its limitations are addressed.

### Final Requirements and Design Outcome: Mobile Self-compassion Intervention

Top-down requirements for the intervention included key components of compassionate mind training (eg, psychoeducation about 3 emotion systems and soothing breathing rhythm exercises) [32] and making use of persuasive design principles such as self-tracking [40]. Bottom-up requirements included addressing common challenges after diagnosis, such as coping with anxiety and regulating information consumption, and tailoring top-down content to the context of cancer by providing applied examples (of peers). Bottom-up requirements related to design and functionality, such as content offered in brief sessions, subtle motivational elements such as progress tracking, and simple navigation and visual design, are in line with other co-design projects of various mobile apps for people with cancer [49-51] and may indicate a common need for a reduced cognitive load when interacting with such apps. Moreover, the final requirements illustrate how we resolved design dilemmas that other eHealth designers may also face, including tunneled versus freely available content [52], offering push notifications without being too intrusive [53], and incorporating automated versus caregiver support [54]. The final design—*Compas-Y*—can be seen as a version of compassionate mind training (top-down input) that is fully adopted for people with cancer (bottom-up input) and also as bottom-up needs that are met with (elements of) compassionate mind training. For example, a bottom-up topic such as *information consumption* (ie, coping with the diagnosis by [excessively] seeking cancer-related information) is integrated with top-down content (eg, acquiring resources [information] as part of our innate drive system), and intervention-specific compassion exercises are offered (eg, observing what emotions are activated when seeking information). Similarly, top-down and bottom-up requirements are implemented at all intervention levels (eg, content, navigation, visual design, and implementation structures).

As there was no existing technology-enabled version of compassionate mind training available, our co-design process also involved adapting a traditional intervention format to a mobile intervention. Similar to most evidence-based psychological interventions, compassionate mind training was originally developed for face-to-face use, using a session-based, didactic training style [55]. However, holding on to this format may not necessarily be fitting or necessary for technology-based interventions and may limit researchers to adopting different means of achieving intervention goals that are unique to mobile- or technology-based interventions [56]. In our adaptation of compassionate mind training, we aimed to make use of the particular characteristics and opportunities of mobile apps (eg, self-tracking and push notifications to facilitate in-the-moment integration of skills; information presented in short texts, videos, audio files, and images; and use of persuasive design strategies and design approaches). Mobile technology not only offers the potential to offer content directed at enhancing users’ own compassion but also to assist with the recognition of distress to model a compassionate response. In *Compas-Y*, this was implemented with a mood tracker that offers feedback adapted to the users’ score (eg, a supportive message when anxiety is high). Other examples include the use of artificial intelligence to recognize the emotional load of text-based diary inputs [57] and the use of sensor-based technologies to track biomarkers related to emotional arousal [58]. With the further development of novel technologies, such opportunities will become more available and affordable and will likely shape further developments in compassion training.

### The Process of Integrating Top-down and Bottom-up Requirements

To achieve the integration of the aforementioned top-down and bottom-up requirements, this study devised and evaluated four co-design tasks: (1) *explore* bottom-up experiences, (2) *reassess* top-down content, (3) *incorporate* bottom-up and top-down input into concrete features and design, and (4) *synergize* bottom-up and top-down input into the intervention context. Overall, the participants evaluated the co-design sessions as valuable and engaging, and the co-design tasks enabled the implementation of all the main requirements into the design. In our co-design study, the 4 tasks enabled us to deal with challenges such as integrating top-down content into the co-design exercises in a balanced way and dealing with conflicting top-down and bottom-up requirements. Dealing with conflicting requirements (and goals, expectations, and power dynamics) is a known challenge in co-design even without introducing top-down requirements [59,60], and working with co-design tasks may offer a helpful way of making divergences explicit. The 4 co-design tasks may be used in the context of established approaches to eHealth development, in which the consideration of theory-based requirements is generally lacking. Indeed, in a recent scoping review of methods used in eHealth development, Kip et al [61] found that very few studies reported on theory-based methods, and the main identified area for improvement for eHealth development models was to add explicit goals and activities aimed at the integration of evidence-based approaches. This study could guide this development, for example, by incorporating a *theoretical framework* as a development phase in existing models and using the 4 co-design tasks to synchronize this with development phases related to bottom-up requirements (eg, the *contextual inquiry* phase in the Centre for eHealth Research Roadmap [29]). Of note, the co-design tasks are likely to need adjustment and re-evaluation in light of the particular characteristics of other co-design projects, for example, when the modality of a design is undefined (eg, offline book or smartphone app) or when external experts rather than researchers represent top-down input during co-design sessions.

Interestingly, the focus on either top-down or bottom-up development of eHealth interventions largely represents differences in the scientific disciplines from which these approaches originate. In behavioral science, developing interventions based on theory and evidence is important not only for developing effective interventions but also for further developing and testing their underlying theories and mechanisms [28]. For example, in intervention mapping, theory-based intervention methods and strategies are selected to meet predefined intervention objectives [27]. By contrast, in design research, developing interventions based on creativity methods without too many predefined objectives is important to allow for innovation and charting of unknown territories. As both approaches have their merit, Schmidt [62] proposed a hybrid interdisciplinary model in which behavioral science can supply evidence-based approaches and design research can offer speculative hypotheses and innovative solutions. Indeed, in our interdisciplinary co-design study, we attempted to bridge both fields by integrating theory-driven (top-down) and contextual (bottom-up) knowledge, as well as by using methodologies from both fields. This resulted in iterative cycles of design and evaluation using (low-fidelity) prototypes while also creating a “final” version of the intervention that could be evaluated in a pre-post hypothesis testing design. Thus, although the underlying principles and quality requirements of these fields may clash at times, we concur that using both generative and analytical approaches offers complementary value in the development of eHealth interventions.

### Strengths and Limitations

This study was strengthened by an extensive co-design development process of 2 years. This allowed for an in-depth exploration of both bottom-up and top-down requirements, as well as thorough field testing and evaluation of co-design tasks. The final intervention is not only a testament to the apparent successful integration of both bottom-up and top-down requirements but also to the adaptation of a traditional intervention to the context of mobile technology. However, this study was limited in several ways. First, the 4 co-design tasks are based only on a single co-design study, and their utility in other contexts remains unclear. A series of co-design studies might have resulted in a different set of tasks based on the challenges that arise across co-design settings. In addition, this study was shaped by predefined objectives based on acquired funding, such as having a working smartphone app after 2 years. Although this limits shifting the agenda to other potential solutions that may arise [63], such objectives can also be seen as a type of top-down requirement (similar to financial constraints) that simply becomes part of the development process. Finally, although this paper focuses on potentially divergent top-down and bottom-up requirements, it does not suggest that there are no divergent requirements *within* top-down (eg, conflicting evidence) or bottom-up (eg, different needs among participants) input (for further discussion of this issue, see the study by Austin et al [46]).

### Conclusions

In the design of eHealth interventions to support people with cancer, an emphasis on evidence-based research needs to be met by taking lived experiences into consideration, and co-design may be used to do so. However, here, the question is, where in the co-design process do the theory and evidence come in? We devised and evaluated 4 co-design tasks to enable the integration of theory and evidence (top-down) requirements with the needs, wishes, and experiences of users and stakeholders (bottom-up). Executed within a series of group-based co-design sessions, the participants evaluated the co-design process as valuable and rewarding. We conclude that the 4 tasks form a helpful preliminary model for integrated top-down–bottom-up eHealth development by making both types of requirements explicit and brought into a shared design conversation. However, the utility of this approach in other co-design contexts (eg, with different financial constraints, design modalities, or project teams) remains unclear. The 4 co-design tasks yielded a final list of requirements, encompassing, for example, the need for tailored, bite-sized, and engaging psychoeducational content on coping with emotions after a cancer diagnosis. The resulting design—*Compas-Y*—is a compassionate mind training app comprising 6 training modules and several supportive functionalities and persuasive elements. This intervention serves as an applied example of how top-down and bottom-up requirements may be integrated into a design, as well as of the adaptation of a traditional intervention format to mobile delivery. Overall, these design and process outcomes serve to further inform technology-enabled compassion training in general and top-down and bottom-up eHealth development in particular, in the context of people with cancer and beyond.

## Appendix

Multimedia Appendix 1 Evaluation questionnaire for the co-design sessions.

Multimedia Appendix 2 Comprehensive overview of app modules, aims, user outcomes, key components, and exercises.

Multimedia Appendix 3 Overview of persuasive design principles incorporated in the intervention Compas-Y.

Multimedia Appendix 4 Video demonstration of the intervention Compas-Y.

Multimedia Appendix 5 Screenshots of the intervention Compas-Y for each final requirement.
