# Supplementary material for: Integrating Top-down and Bottom-up Requirements in eHealth Development: The Case of a Mobile Self-compassion Intervention for People With Newly Diagnosed Cancer
Source: JMIR Cancer. 2022 Aug 1;8(3):e37502. doi: 10.2196/37502 (PMC9379787; doi:10.2196/37502)
Supplement: Multimedia Appendix 1 [file cancer_v8i3e37502_app1.pdf]

Session evaluation form

Below you will find a few questions regarding how you experienced the session. Thank you for your input!

Please mark the answer that best represents your experience:	Bad/not much	Mediocre/ a little	Okay/ somewhat	Good/ plenty	Great/ very much	*Elaborate: (optional)*
1. What did you think of the content of the session?						
2. To what extent did the session match your energy level?						
3. To what extent did you find the session inspiring?						
4. To what extent did you learn something new during the session?						
5. Did the workshop sufficiently align with your knowledge and expertise?						
6. To what extent do you feel involved with developing a self-compassion app for people with cancer?						
7. Do you feel like you have influence over the design of the self-compassion app?						
8. How did you experience collaborating with different people (people with cancer, nurses, researchers, designers)?						

What did you find the nicest, best or most valuable about the session?

………

………

Did the session meet your expectations?

………

………

Do you have any suggestions for improvement?

………

………
