# Supplementary material for: Integrating Top-down and Bottom-up Requirements in eHealth Development: The Case of a Mobile Self-compassion Intervention for People With Newly Diagnosed Cancer
Source: JMIR Cancer. 2022 Aug 1;8(3):e37502. doi: 10.2196/37502 (PMC9379787; doi:10.2196/37502)
Supplement: Multimedia Appendix 2 [file cancer_v8i3e37502_app2.pdf]

Comprehensive overview of app modules, aims, user outcomes, key components and exercises.

Module	Aims	User outcomes	Key components and exercises
1. Introduction to the app and self-compassion	- Provide recognition for emotional aspects of cancer diagnosis and introduce app as a self-support tool<br>- Introduce self-compassion and enable self-reflection about self-compassion	- Feeling recognized in the emotional challenges that come with a cancer diagnosis<br>- Having a clear picture of the app and what personal needs and motivations for use are<br>- Having insight into current experience and extent of self-compassion	- Psycho-education about self-compassion<br>- Exercises in mindful awareness and soothing breathing rhythm<br>- Exercise in recognizing (brief) positive experiences throughout the day
2. Emotions in the context of cancer	- De-shaming of emotions by explaining and practicing with three emotion systems<br>- Strengthening soothing system by finding moments of calm and connection, practicing imagery<br>- Balancing the drive system based on the example of searching for information<br>- Balancing the threat system based on the example of anxiety and cancer-related fears	- Increased understanding and recognition of emotional experiences<br>- Tools to accept and self-regulate emotions (and anxiety in particular) by mindful awareness and self-soothing<br>- Ability to find balance between gathering helpful resources (e.g. information about diagnosis) and not getting overwhelmed by them	- Psycho-education about three emotion systems<br>- Soothing breathing rhythm exercise with imagery<br>- Compassionate information seeking; finding resources based on own needs<br>- Psycho-education about anxiety and practicing to recognize and allow anxiety
3. Self-compassion and self-criticism	- Provide insights in the forms and roles of self-criticism and how this applies to the context of cancer<br>- Getting familiar with inner critic, and what its functions are<br>- Provide insights in the forms and roles of self-compassion and how it applies to the context of	- Recognizing the words, tone of voice, triggers of self-criticism<br>- Learn compassionate alternatives of self-relating<br>- Having insight into the difference between self-criticism and self-compassion and having the tools to be more self-compassionate	- Psycho-education about self-compassion and self-criticism<br>- Imagery exercises about compassionate self and inner critic<br>- Soothing breathing exercise with compassionate friend<br>- Self-compassion expressive writing exercise

	cancer<br>- Getting familiar with compassionate self, and how it is helpful		
4. Taking care of your body	- Learn to have compassion for (experiences related to) the body: including all (non)functional, ill, and (un)attractive parts<br>- Learn to practice lifestyle behaviors with compassion and compassionate goal-setting<br>- Provide recognition for (and stimulate communication about) the influence of cancer on sexuality and intimacy, and discuss how to meet needs with compassion	- Increased connection and reduced alienation from body<br>- Insight into compassionate vs. fear-based goals and increased self-care behaviors<br>- Allowance and recognition of and compassion for sexual and intimacy experiences in the context of cancer (increased tools to care for needs/knowing where to find additional resources)	- Soothing breathing rhythm-based compassionate body scan<br>- Psycho-education and exercises about the difference between compassionate motivation and self-correction and self-critical motivation or attacking<br>- Psycho-education about compassion for own needs in the context of sexuality and intimacy
5. The people around you	- Awareness and practice of giving and receiving compassion to/from others and related obstacles to compassion<br>- Learn to set boundaries and to ask and accept for help<br>- Provide recognition for loneliness and practicing (awareness of) connection with others	- Being able to set boundaries in social contact based on own needs (and needs of others)<br>- Being able to ask for and receive help based on own needs<br>- Recognizing and allowing loneliness, feeling more connection with others	-Psycho-education about the three flows of compassion<br>- Soothing breathing rhythm-based loving-kindness meditation<br>- Setting boundaries and asking for help based on compassion for own needs
6. Continuing with resilience	- Cultivate resources for well-being and resilience by practicing gratitude, savoring, recognizing strengths, recognizing values<br>- Reflect on the past 6 weeks: what has been learned, what was the most beneficial, what was not<br>- Build on personal strengths to maintain	- Increased experience and practice of gratitude<br>- Ability to sense and savor pleasant experiences<br>- Awareness and appreciation of personal strengths<br>- Ability to build on strengths to maintain self-compassion practice	- Psycho-education and exercises about positive psychology: gratitude, wellbeing, savoring, personal strengths<br>- Reflection on self-compassion practice and how to continue<br>- Soothing breathing rhythm meditation (with focus on tone of voice,

	practices (as desired) post-intervention		posture etc.)
Supportive functionalities	Aims	Outcome user	Key element
Visual overview of modules	- Provide overview of which modules are available, user progress and completion of modules	- Insight into own progress and available modules	Visual element central to the homepage (compass) that depicts the 6 modules
Mood tracker	- Increased awareness of emotional experiences based on self-monitoring as a basis for self-soothing and compassionate actions<br>- Linking the supportive functionalities of the app with the module content	- Increased awareness of emotional experiences and how they relate to day-to-day experiences<br>- Recognition of and suggestions for practicing with emotions	- Mood tracking with automated feedback based on three emotion systems
Favorite exercises	- Provide a personalized experience for the user where they can easily access the parts of the app that they prefer, which in turn would lead to increased engagement	- An app that is tailored to own needs and preferences, thereby creating a more relevant intervention	- Marking exercises as favorite within the modules, which then appear in the users' personal list of favorites
Light of the day	- Increased attention for (brief) positive experiences throughout the day, which contributes to wellbeing	- Experiencing that there is "more to life than cancer", that hardship and positivity can co-exist: finding (brief) positive experiences throughout the day	- Exercise where user inputs a positive experience of their day, supported by examples
Practical information	- Providing reliable links where users can find more information about different aspects of living with cancer, as well as additional self-compassion resources<br>- Providing background information about how, by whom and based on what the app was developed<br>- Providing information about privacy<br>- Providing contact information for technical and content-related questions	- Having a reliable, pre-sorted and structured list with information sources, reducing the need and effort to (over)use a search engine to find suitable information<br>- Having transparent background information about the app, personal data and what to do in case of questions	- List with weblinks, each with descriptions
Push notifications	- Provide daily inspiration and brief exercises that aid with the integration of self-	- Integration of moments of inspiration, mindful	- Daily messages, with an option to reduce the

	compassion practices throughout the day<br>- Remind the user of the possibility to use the app in an indirect, inspirational manner	awareness and gratitude into daily life<br>- Being reminded of the possibility to use the app (also helpful in case of symptoms of forgetfulness and reduced focus)	frequency or turn messages off
