# Supplementary material for: Integrating Top-down and Bottom-up Requirements in eHealth Development: The Case of a Mobile Self-compassion Intervention for People With Newly Diagnosed Cancer
Source: JMIR Cancer. 2022 Aug 1;8(3):e37502. doi: 10.2196/37502 (PMC9379787; doi:10.2196/37502)
Supplement: Multimedia Appendix 3 [file cancer_v8i3e37502_app3.pdf]

Overview of persuasive design principles incorporated in the intervention *Compas-Y*.*

Principle	Implementation
Primary task support	
Reduction	Providing examples of self-compassionate responding in quotes, exercises, example behaviors
Tunneling	Sequential structure in modules, e.g. starting with basic mindfulness exercises before visualization
Tailoring	Content is fully tailored to context of cancer, e.g. with examples from peers and frequently experienced challenges
Personalization	Limited personalization in the form of automated feedback based on responses, options to save and skip exercises
Self-monitoring	Tracking progress across modules, monitoring mood with mood tracker
Simulation	None (other than examples of differences in emotions following self-compassionate responding rather than self-critical responding)
Rehearsal	Exercises are repeated and elaborated upon multiple times (e.g. each meditation exercise builds upon soothing rhythm breathing)
Dialogue support	
Praise	Minimal, but included in automated feedback
Rewards	Light of the day symbol lights up
Reminders	Push notifications with inspirational quotes or small exercises
Suggestion	Automated feedback based on user input
Similarity	-
Liking	Minimal and soothing visible design
Social role	No particular social role (other than automated feedback)
System credibility support	
Trustworthiness	Information about development process of the app
Expertise	Selected links with information about cancer, expertise of development team
Surface credibility	Information about organizations and people behind app
Authority	Universities, hospitals and funder are mentioned
Third-party endorsements	Funder (KWF) is mentioned
Verifiability	Links to other websites (incl. privacy regulation of the university)
Social support	
Social learning	Limited, by providing quotes of peers about their tips and experiences
Social comparison	“”
Normative influence	“”
Social facilitation	“”
Cooperation	-
Competition	-
Recognition	-

* Note: Persuasive design elements (for an overview see [40]) are predominantly incorporated through primary task support (e.g. tailoring of content to situation of cancer, self-monitoring of progress and of mood, rehearsal of soothing rhythm breathing exercises). Furthermore, principles related to dialogue support are embedded (e.g. suggestion through automated feedback, reward in the form of a sun turning yellow after adding a 'light of the day') as well as system credibility support (e.g. third-party endorsement by funder, portraying trustworthiness with background information about app development). Social support principles such as social cooperation, competition and recognition are not embedded given the private nature of the app. In line with the requirements, personalization is relatively minimal (e.g. option to mark exercises as favorite, yet all users are offered the same content) as are rewards (e.g. no badges or points to be earned).
