# Supplementary material for: Integrating Top-down and Bottom-up Requirements in eHealth Development: The Case of a Mobile Self-compassion Intervention for People With Newly Diagnosed Cancer
Source: JMIR Cancer. 2022 Aug 1;8(3):e37502. doi: 10.2196/37502 (PMC9379787; doi:10.2196/37502)
Supplement: Multimedia Appendix 5 [file cancer_v8i3e37502_app5.pdf]

Screenshots of the intervention *Compas-Y* for each final requirement.*

<table>
<tr><th>Req.</th><th>Screenshot</th><th>Description of screenshot</th></tr>
<tr><td>1</td><td>
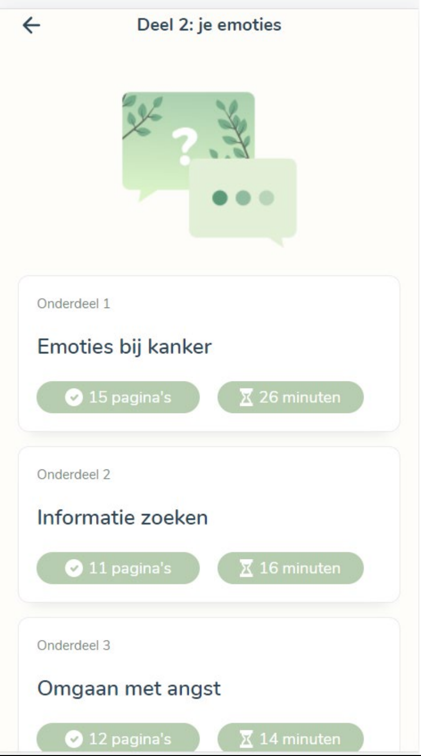

</td><td>These three submodules address different bottom-up topics</td></tr>
<tr><td>2</td><td>
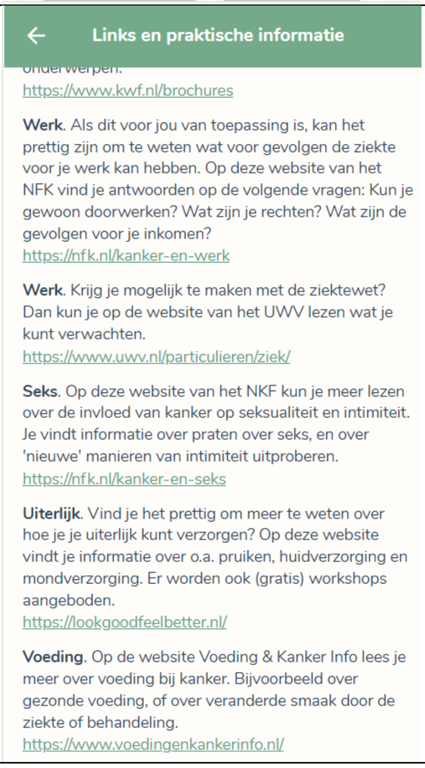

</td><td>This information page contains selected weblinks with practical cancer-related information</td></tr>
</table>

3	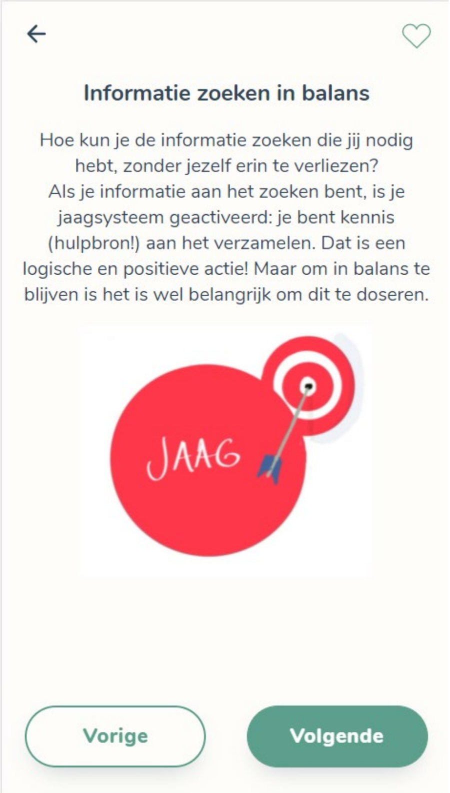	This psycho-education about the drive system is tailored to the context of cancer (on the topic of seeking information)
4	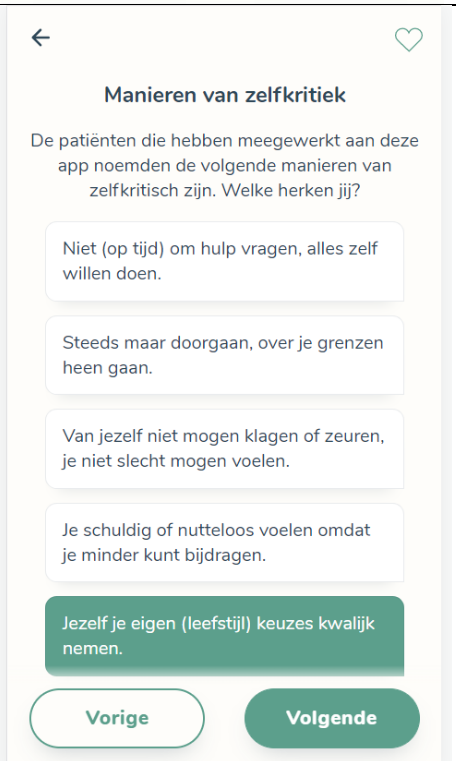	This reflective exercise is about self-criticism tailored to the context of cancer by using examples of peers

5	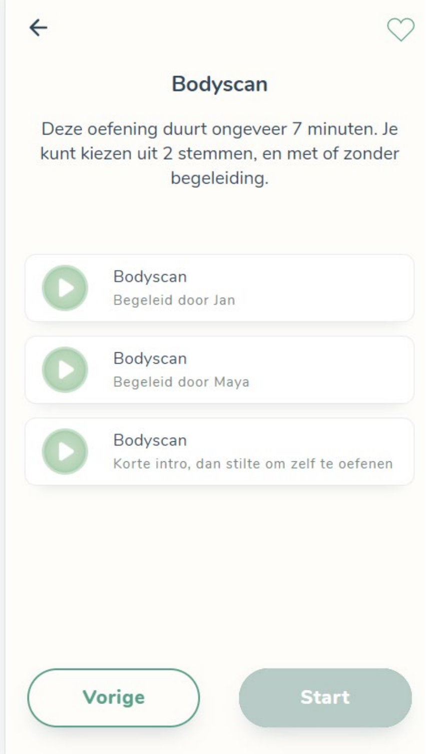	This body scan exercise is brief and is audio-guided in a down-to-earth, practical manner
6	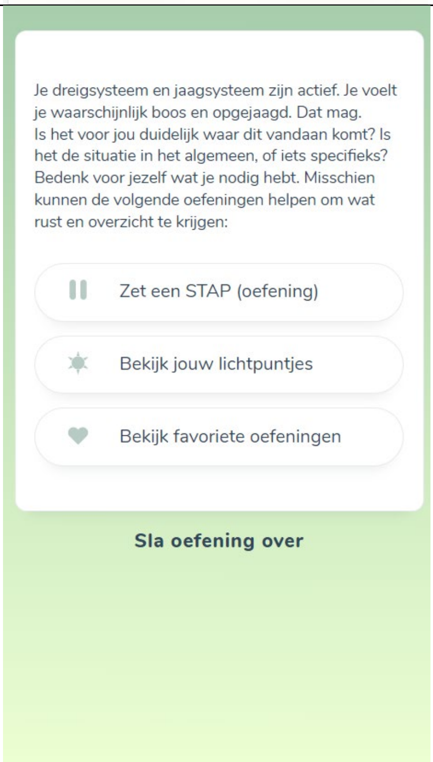	This automated feedback text is based on mood tracker input and utilizes compassionate language while also offering suggestions for exercises

<table>
<tr><td>7</td><td>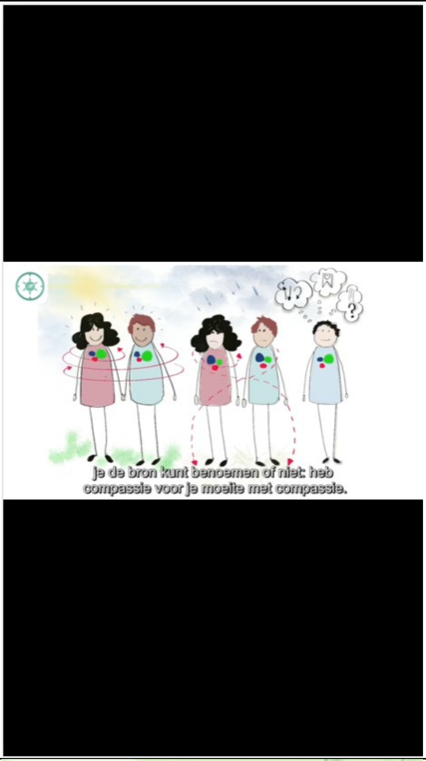
</td><td>This video conveys psycho-education about the flows of compassion (compassion for/from others)</td></tr>
<tr><td>8</td><td>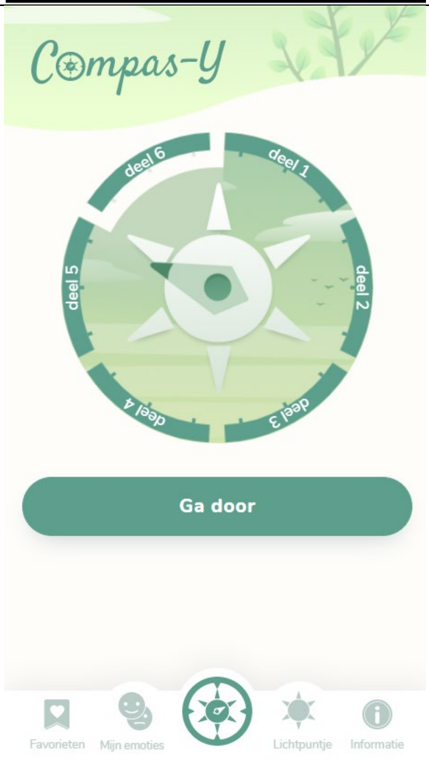
</td><td>This compass symbol shows a visual reward (a new part of an incomplete image in the middle of the compass appears upon completing a module)</td></tr>
</table>

<table>
<tr>
<td>9</td>
<td>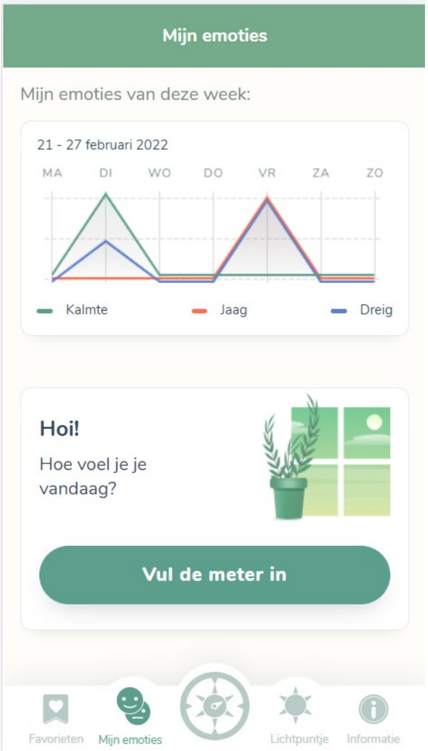
</td>
<td>This mood tracker graph shows mood progression over time</td>
</tr>
<tr>
<td>10</td>
<td>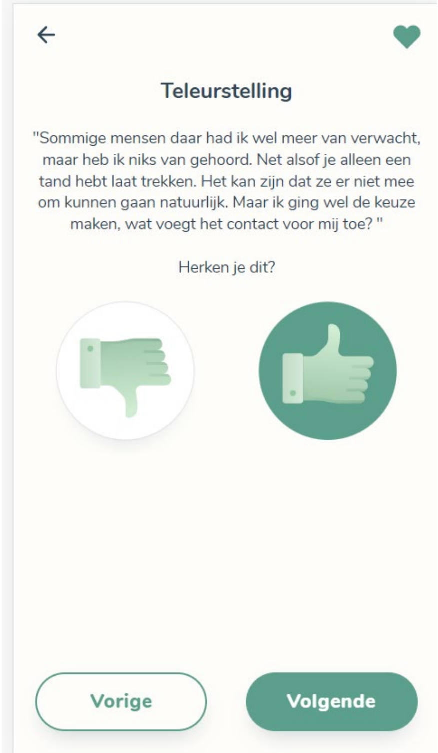
</td>
<td>This quote offers indirect experiences from a peer in the context of the module theme</td>
</tr>
</table>

11	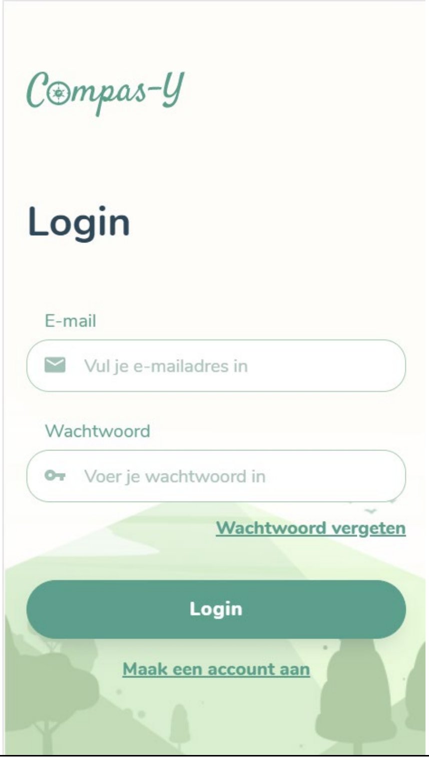	This simple on-boarding requires creating an account on registration, while staying logged-in for subsequent sessions
12-14	Not applicable	Not applicable
15	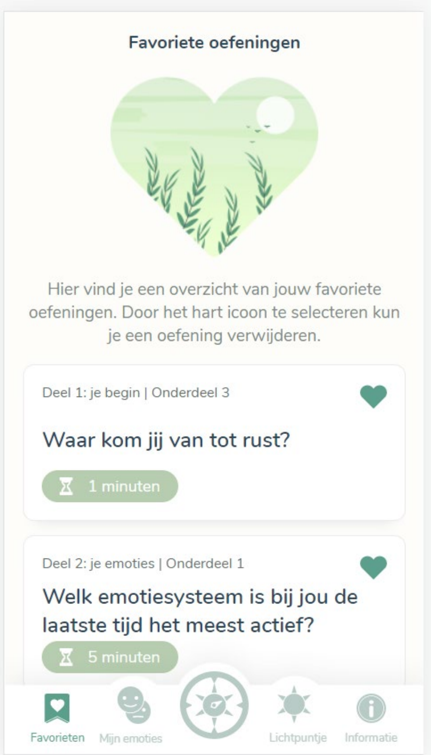	This list with favorites displays exercises that were marked in the modules

*The numbered requirements in the first column correspond to final requirements of Table 2.
